# Testing weight-based conditional discrimination in Goffin’s cockatoos, *Cacatua goffiniana*

**DOI:** 10.1371/journal.pone.0338604

**Published:** 2025-12-19

**Authors:** Poppy J. Lambert, Antonia Rippel-Rachle, Alice M. I. Auersperg

**Affiliations:** Messerli Research Institute, University of Veterinary Medicine Vienna, Vienna, Austria; University of Tuscia, ITALY

## Abstract

Discrimination learning tasks are a method for investigating species’ perception of and associative learning with a particular stimulus. Goffin’s cockatoos previously required surprisingly few trials to differentiate objects based on weight alone in a simple discrimination task, outperforming primates in other weight discrimination setups. Nevertheless, it was unclear whether the difference in performance was largely based on ability or differences in experimental procedures. Therefore, we tested a group of Goffin’s cockatoos on a weight-based conditional discrimination, like a task previously used with chimpanzees. Similar to the chimpanzees (compared to their first 15 sessions), none of our subjects reached above-chance levels of performance in the present study. The contrasting performance of our cockatoos in the two weight discrimination studies are in line with the idea that conditional discriminations are more cognitively demanding than simple discriminations. Our results do not support the notion of a distinct difference between birds and primates in their arbitrary discrimination learning abilities with weight cues. However, further research on this question (we suggest with simple discrimination formats) would be necessary.

## Introduction

Classical discrimination learning tasks examine perception and associative learning. They can be used, for example, to investigate species’ learning abilities with different cue types (e.g., [1]), or to compare the cognitive abilities within or between groups (e.g., [2–4]). Classical discrimination learning in nonhuman animals with weight (proprioceptive) cues has been somewhat understudied, compared to discrimination learning with other forms of information. There are only a handful of classical discrimination studies (i.e., with arbitrary relationships between stimulus and reward) involving exclusively weight cues. These exist across a few different animal groups: octopus (*Octopus vulgaris*) [5], rats (*Rattus norvegicus*) [6,7], Goffin’s cockatoos (*Cacatua goffiniana*) [8], and primates, including capuchin monkeys (*Cebus capucinus*), macaques (*Macacus cynomolgos*) and spider monkeys (*Ateles geoffroyi*) [9], gorillas (*Gorilla gorilla*), orangutans (*Pongo pygmaeus*), and bonobos (*Pan paniscus*) [10], and chimpanzees (*Pan troglodytes*) [11,12]. The recognition and use of weight information has been investigated in various other specific contexts (primarily within primate and large-brained avian species), such as in tool-use (e.g., [13–15]), physical problem-solving (e.g., [16,17]), and inferential reasoning (e.g., [12,18–23]). Nevertheless, more basic studies focusing solely on weight discrimination, to enable comparisons between the abilities of distantly related species, are presently required in order to achieve a more meaningful understanding of the evolution of decision-making with weight information in animals.

For example, it is interesting to ask how species might have diverged with respect to their associative learning capabilities, particularly in light of differences in lifestyle and in the ecological relevance of different object properties [4,8,12]. The results of recent research offer the suggestion that birds – at least Goffin’s cockatoos – might have a greater ability to learn an arbitrary discrimination based on solely weight information, given that subjects learnt a weight discrimination in around 60 trials on average [8], compared to primates, which have required hundreds of trials to meet experimental criteria (see: [9–12]). This stark difference is interesting considering that, similarly to Goffin’s cockatoos (e.g., [24–28]), the great apes are known to engage in high levels of object manipulations and tool use (e.g., [29,30]). A possible reason for a difference in associative learning abilities with weight cues between the two groups might be a difference in sensitivity to the object property. In other words, there could be differences in top-down (internal) selective attention mechanisms at work (e.g., see [31,32]) with a higher degree of attention to weight information in birds perhaps emerging from the energetic demands of travelling by flying [33] (but see [34]).

However, the discrimination task designed by Lambert and colleagues [8], in which Goffin’s cockatoos (also referred to as ‘Goffins’) quickly learnt to discriminate objects by weight, is the only weight discrimination paradigm (of the arbitrary discrimination learning paradigms we discuss; see [13,14] for examples in a tool-use context) to involve the *mandatory* sampling of both object types, light and heavy, within a trial. Specifically, the birds were asked to pick up and move each of the two differently weighted objects before they could make a choice of one object to give to the experimenter. This first step might have enhanced learning about the respective weights or highlighted the object property that was important to the discrimination, promoting overall acquisition of the task relative to other methods.

Only one weight-based arbitrary discrimination learning study has involved a conditional discrimination task (with chimpanzees: [12]), rather than a ‘simple’ discrimination task format [5–11]. In a conditional discrimination task, the rewarded response depends upon the type of stimulus presented (on a trial), requiring subjects to learn a set of rules such as, ‘if A, then X; if B, then Y’. Conditional discriminations are considered more difficult to learn than simple discriminations, in which one choice or response is always rewarded (e.g., [35,36, Experience phase]. The task used for chimpanzees, a weight sorting task, required subjects to learn to place the weighted object presented on a trial into the correct tray, depending on its weight type, in order to receive a reward [12, Experiment 9]. The rules to be learned were ‘if the object is heavy, put it in the blue/right tray; if the object is light, put it in the orange/left tray’. The chimpanzees required an average of 895.2 trials (a range of 390–1562 trials; one individual did not learn the discrimination) to learn to sort the weighted objects to a 90% or higher degree of accuracy.

One unanswered question is how Goffin’s cockatoos would perform on a conditional discrimination task involving a weight stimulus. They have already demonstrated their ability to pay attention to this physical property [8,34]. Given the ecological relevance of proprioceptive information to the species, it is possible they could learn the contingencies of this conditional discrimination task when they have failed or found it difficult to learn contingencies in contexts that are less ‘physical’ (e.g., matching- and non-matching-to-sample on a touchscreen task [37]). A second question is whether Goffin’s cockatoos still outperform primates on a weight discrimination learning task when tested with a method that is comparable.

In order to answer these questions, we designed a conditional discrimination task as a close replication of the ‘weight sorting’ task Povinelli [12] used with chimpanzees, with which to test Goffin’s cockatoos. In our task, subjects had to learn to place the weighted object in the correctly coloured tray which could be located to the left or right of the object. We made this change to methodology as compared to the original study [12] to try avoid our subjects developing a tendency to take the differently weighted objects to different sides (and potentially impacting performances in future studies), and because we learned from previous studies that the Goffins have a tendency for side biases [28,38,39]. Furthermore, we observed how overall side biases in the chimpanzees in their first 15 sessions [12] (trial-by-trial data was kindly provided directly by the author) led to distinctly contrasting levels of success between trials with the heavy and light object, given that one constant colour-location combination was correct for each of the two object types.

Our first hypothesis, given the understanding that conditional discriminations are more difficult than simple discriminations (e.g., [35,36, Experience phase]), was that our subjects would take longer to learn the present task than they took to learn the previous weight-based discrimination task [8]. In other words, we predicted that success rate on the conditional discrimination would be lower, although we did expect our subjects to learn the task’s sorting rules within the time-frame of this experiment (see Methods). If we found that they performed as well as they did previously on the simple discrimination task (i.e., if there was no significant difference between success rates on the two tasks), this could suggest that weight as a stimulus, or proprioceptive or physical information broadly, is extremely salient to Goffin’s cockatoos, meaning that individuals can learn even complicated contingencies easily in this context.

We also hypothesised that there would be a difference in learning rates shown by subjects between trials where the light weight was presented for sorting and trials where the heavy weight was presented. For example, rats learnt more quickly when light was the correct choice [6], as did Goffin’s cockatoos in the simple weight discrimination task [8]. This observation can be explained by a principle of, or preference towards, ‘least effort’ [6]: if there is an initial preference to move the light option, as it requires less effort than moving the heavy, then subjects would more quickly assimilate a rule where choosing the light option leads to a reward. On the other hand, when looking at the group data for the chimpanzees in their sorting study [12], we see a higher performance overall in trials with the heavy weight (although in this case this likely reflects in most part a group-level overall side bias towards the right). Nonetheless, perception and learning theory supports the idea that learning rates would be higher for the heavy object. This is because handling a heavier weight requires greater muscle tension (e.g., [40,41]), therefore creating a more intense stimulus, and stimulus intensity positively influences learning rate [42]. As such, we did not have a prediction on the direction we would see a difference between performance on light and heavy trials.

Lastly, if the large difference between Goffins and primate species in the number of trials required to reach experimental criteria in previous weight discrimination tasks [8–12] is primarily due to a difference in associative learning abilities with weight in birds and primates, we predicted that Goffin’s cockatoos would have a higher success rate on the weight-sorting task than the chimpanzees of Povinelli’s weight sorting study [12] (with some caveats to take into consideration; see Discussion). If instead the difference in performances can be largely attributed to the paradigm used by Lambert and colleagues [8], we predicted that the learning rates of Goffin’s on the present task would not be meaningfully different than those demonstrated by the chimpanzees.

## Method

### Ethics statement

The study was discussed and approved (ETK-145/09/2020) by the institutional ethics and animal welfare committee of the University of Veterinary Medicine Vienna in accordance with GSP guidelines and national legislation.

### Subjects and housing

16 captive-born and hand-reared Goffin’s cockatoos participated in this study: nine males (one 3-year-old sub-adult and eight adults, aged between 10 and 13 years old) and seven females (two 3-year-old sub-adults and five adults, aged between 10 and 13 years old). All of the birds have CITES certificates and are registered with the district’s animal welfare bureau (Bezirkshauptmannschaft, Schmiedgasse 4–6, A-3100, St. Pölten, Austria). The subjects were housed together in a single social flock at the Goffin Lab Goldegg, in Lower Austria. The aviary consists of an indoor and outdoor area (indoors: 45m^2^ ground space, 3–6m high wall to gable; outdoors: 150m^2^ ground space, 3–4.5m high). The access to the outdoor area is dependent on outside temperatures and the inside aviary is enriched with branches, baths and parrot toys. The birds receive fresh food (vegetables and fruit) twice a day, and a dried lunch with added supplements. They always have access to water. Testing was carried out in an adjoining room where subjects were visually isolated from the rest of the flock. Participation in the task was voluntary; subjects are called to the testing room as an invitation to participate and later in the testing room they can also choose not to engage with the task. All subjects had previous experience with a single weight-based discrimination task [8].

### Apparatus

The weighted objects were grey spheres, approximately 15 mm in diameter, hand-crafted from hard-baked modelling clay (® Fimo). The heavy versions (23g) contained a lead fishing weight inside and the light versions (3g) contained a compressed cotton ball of the same size. These items did not move around or rattle inside the clay ball. The weights of the heavy and light objects were very close in weight to those used in a previous weight discrimination study (4g and 23g) [8]. Multiple objects were made so that they could be replaced immediately if the birds scratched or broke them during testing, ensuring that there were never consistent visual marks to distinguish heavy from light objects.

**Fig 1:**
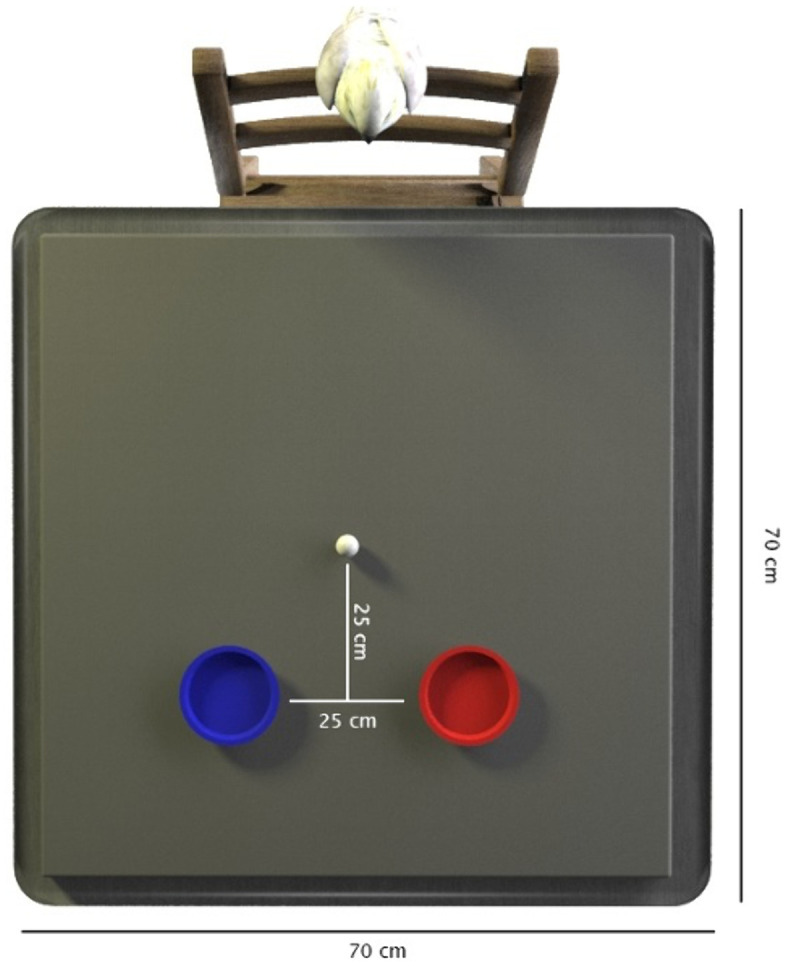
The placement of experimental materials for a trial. The dimensions of the table and the distances between trays and objects are depicted, with a subject waiting on a chair opposite the experimental materials and experimenter (not pictured).

Other experimental materials included two trays made of thin plastic, which were 10 cm in diameter and approximately 1 cm high at the edge of the tray, covered in blue or red felt, and a piece of foam, about 3 cm high, covered in white cotton. This foam cushion was placed on top of the table (which measured 70 by 70 cm; the cushion had approximately the same dimensions) and was for the purpose of reducing the noises made when the objects were dropped, thus minimising the difference between any auditory cues provided by the differently weighted objects. In between trials the birds sat on the backrest of a wooden chair positioned by the table. Fig 1 shows the placement of our experimental materials for a trial.

### Procedure

We assigned individuals into one of two groups with opposite ‘sorting’ rules – i.e., the subject was either rewarded for placing the light object into the red tray (Group 1), versus rewarded for placing light into blue (Group 2), with the opposite contingencies for the heavy object – to account for a possible colour preference across subjects. We controlled for sex and age between the two groups as far as possible (see Table A in the Supporting information for details). In the week preceding the beginning of testing, the birds were habituated to walking on the cushion on the table. This involved placing nuts and seeds on the cushion, without any other experimental materials present, to encourage the birds to step up from a chair onto the cushion and walk across it; no subject required more than three such sessions in order to show no signs of neophobia or discomfort. The subjects already had experience placing objects into trays as part of a previous experimental task [8] for which the command ‘give me’ was used, so no training with this command was required for this study. We note that in the previous task the experimenter used this verbal cue simultaneously with pointing (into the choice tray), and in the present study the experimenter just used the verbal cue – unless the subject did not quickly pick up the object to move it into a tray (see below). Subjects were not introduced to any task contingencies before the first trial.

Each trial involved a single heavy or light object. Object type was pseudorandomised across trials and the left-right location of the blue and red trays was also pseudorandomised across trials. A trial could have one of four possible combinations of weight type and tray placement – for example, a trial where the heavy object is presented for sorting and the blue tray is on the left (and red on right), or the light object is presented and the red tray is on the left, and so on. No object type or tray placement occurred for more than three consecutive trials within a session. Overall, subjects received approximately equal numbers of trials with the heavy and light weight, and the four trial types in terms of weight-tray location combinations. The identity of all trials can be found in the S1 Dataset. Subjects were tested in blocks of ten trials – constituting one session – and were given a maximum of one session per day.

We chose to set a maximum number of 200 trials for testing the Goffin’s cockatoos on the task. This upper limit was informed by two main considerations. Firstly, it is more than three times the average number of trials subjects required to meet our criterion in the simple discrimination (an average of 60.6 trials; subject range 30–88, excluding one individual who did not learn within 100 trials [8]), and also greater than the number of trials (140 or less) our subjects required in this first discrimination to achieve a stricter criterion of 45/50 correct (as used by [12]). Therefore, we considered it sufficient to detect any within-individual differences in learning rates between the two tasks (weight choice and weight sorting) in our Goffin subjects. Secondly, it is of a similar order to the number of trials some other species have required in comparable active-choice conditional discrimination studies (e.g., [3,43]), so we viewed it as providing a reasonable opportunity to our subjects for forming associations between stimulus and response, i.e., learning the contingencies of the task.

**Fig 2:**
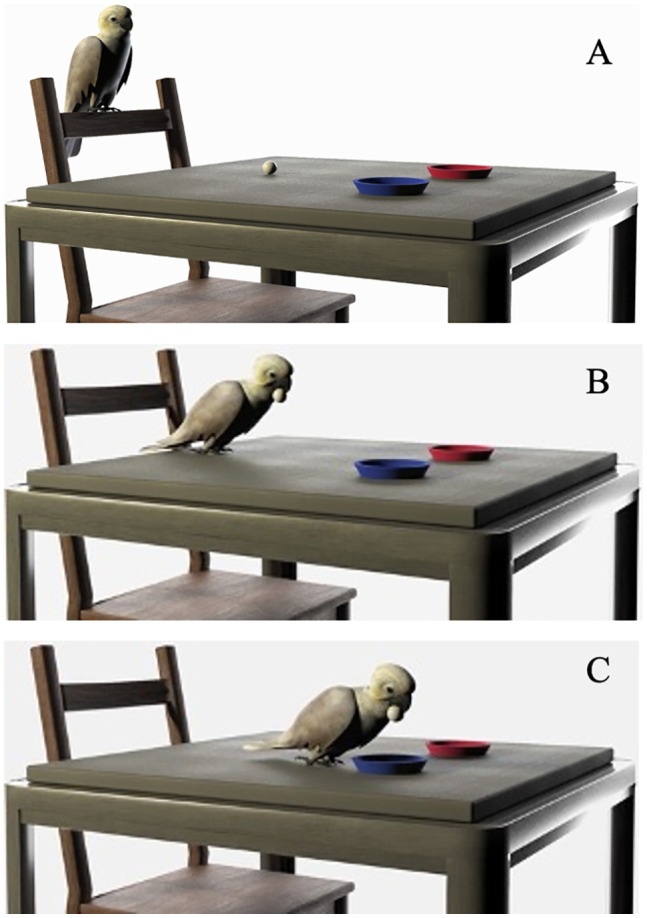
A visualisation of a subject participating in a single trial. **(A)** The bird’s starting position. **(B)** The bird picking up the object from where it has been placed by the experimenter. **(C)** The bird making a choice by placing the object in one tray.

To set up a trial, the experimenter placed the two trays on the table, about 25 cm apart, and then placed the weighted object equidistant to both trays and between the subject and the trays (see Fig 1), whilst the subject waited on the chair opposite. The experimenter instructed the subject to begin a trial with the verbal cue ‘give me’. To complete a trial, the bird stepped from the backrest of the chair onto the table, picked up the object in their beak, walked forwards and placed the object onto one of the trays (see Fig 2). If a bird did not begin a trial, the experimenter further prompted them by pointing between the trays and repeating the verbal cue. The subject’s choice was counted as the first contact of the object made with either of the coloured trays. If they chose correctly, they were immediately given a small piece (about an 1/8^th^) of cashew as a reward. However, if they chose the incorrect tray, the experimenter said ‘nein’. In either case the experimenter asked the bird to return to the waiting position and removed the weighted object for the next trial (and the two trays if their position was to be changed). During testing, the experimenter wore mirrored sunglasses, faced centrally between the two trays and made minimal movements to avoid cuing the birds. During testing the birds’ responses were live-coded. The trial videos have been reviewed to check for any possible cueing.

### Analysis

We ran a series of generalised linear mixed models (GLMMs), all with binomial error structure and including subjects’ success data from trials (0/1) as the response variable. All analyses were carried out in R (version 4.4.2) [44] using the function ‘glmer’ from the lme4 package (version: 1.1–35.5) and we used the package ‘ggplot2’ for generating figures (see Supporting information for further details on model implementation). In our analyses we compared full models to respective null models to avoid the risk of increased type 1 error due to multiple testing. We z-transformed the continuous variables of trial number and session number in all analyses and dummy coded and centred factors (group, weight, task) where appropriate. In our models we included the random intercept of subject to avoid pseudo-replication and also a combined factor of session and subject (‘dayID’) to account for variation in, e.g., motivation on each day. We included all theoretically identifiable random slopes to balance type I and type II errors. We removed correlations of random slopes and random intercepts if the majority of correlations appeared unidentifiable [45] or if any resulted in a non-numerical output.

With our first model we investigated the performance of Goffin’s cockatoos on the sorting task. In the model we included the predictors of trial number, session number and weight type presented on the trial (hereafter ‘weight type’). We included these terms as a three-way interaction because we hypothesised that it would be easier to learn the sorting rule for one weight type (heavy/light) over the other (see [6,8,12]). Group was further included as a predictor variable to control or account for overall differences in performance between the two groups with opposite sorting rules, which could arise from potential colour preferences. Five subjects ceased participating before reaching 200 trials and a further subject was accidently cued in the third session, so only data from this subject’s first two sessions are included in the analysis (n: 16; total number of observations: 2310) (see Table 1 for details on how many trials each Goffin subject contributed to the dataset).

**Table 1:** Trials contributed by Goffin subjects to the weight sorting dataset.

Subject (Goffin)	Number of trials included in Models 1a/b
Titus	20
Dolittle	40
Muki	50
Zozo	50
Konrad	70
Heidi	80
All other subjects (n = 10: Figaro, Fini, Irene, Jane, Kiwi, Mayday, Moneypenny, Muppet, Olympia, Pipin)	200

With one version of the model (model 1a), we tested if the Goffins’ overall group-level performance was significantly different from chance level; the estimate of the intercept was of interest here. In model 1a the predictors of weight and group were additionally dummy coded and centred. With the second version of the model (1b; weight and group not dummy coded), we investigated whether there was a statistically significant difference in subjects’ success rate between heavy and light trials; the weight term was dropped from the null model.

With our second model we investigated the influence of task type (the previous weight ‘choice’ task and the present weight ‘sorting’ task) on success rate in Goffin subjects. Task was included in interaction with our other predictor term, cumulative trial number; task was dropped from the null model. We chose to include cumulative trial number rather than trial number and session number (as for models 1a/b), as we were not interested in how the gradient of learning *within* sessions might have differed between the two tasks, only how *overall* learning rates might have differed. For this within-subject comparison necessarily only subjects which were in the ‘test-only’ group in the weight choice task (see [8]) were included in the analysis (n:8; total number of observations: 1460). We matched the number of trials included for each subject in each task to the minimum number of trials they participated in within either task (see Table 2).

**Table 2:** Matched trials contributed by Goffin subjects to the task comparison dataset.

Subject (Goffin)	Matched number of trials^a^ included in Model 2)
Dolittle	40
Konrad	70
Heidi	80
Mayday	80
Jane	100
Muppet	110
Pipin	110
Moneypenny	140

^a^the number reported represents one task, so the total number of trials contributed by the individual to the analysis is double the number stated here.

Lastly, with our third model we examined the performance of chimpanzees on the weight sorting task [12] over their first 15 sessions (with data kindly provided by the author) (n:7; total number of observations: 1050). Given the methodological difference between our task and that designed by Povinelli [12] with regards to tray location (as described in the introduction) and further the preliminary training the chimpanzees received but we did not provide the Goffins, we viewed a direct statistical comparison of the performances of both species inappropriate. Model 3 was constructed like model 1, but with no control predictor of group as there were no groups in the study with chimpanzees [12]. We otherwise used the same approach as described for model 1.

## Results

**Fig 3:**
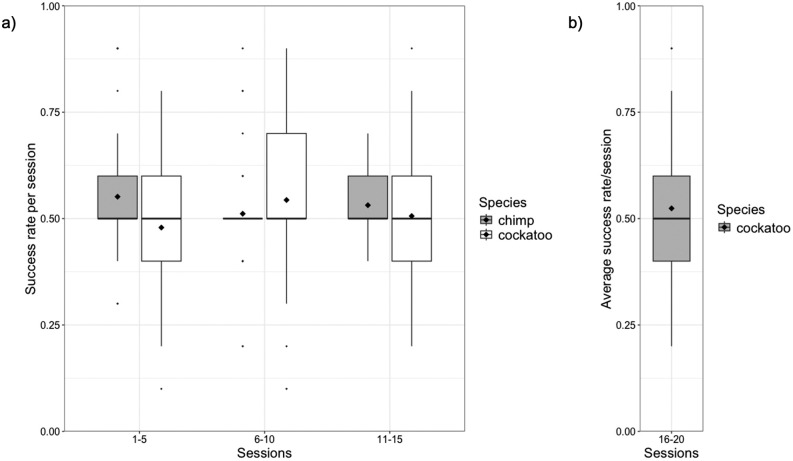
(a and b) Success rate for Goffins and chimpanzees in their respective weight sorting tasks. The success rate for Goffin’s cockatoos (present study; grey bar plots) and chimpanzees ([12]; white bar plots), summarised in three blocks of five sessions (note that each observation represents one session from one subject). The mean value is indicated by the diamond.

Goffin subjects did not sort the rewarded weight into the correct tray above chance levels (see data depicted in Fig 3) (model 1a; intercept estimate: 0.037, s.e. ± 0.043, z = 0.849, p = 0.396). At a group level, subjects had an overall estimated probability of being correct of 50.9% (see Table B in Supporting information for individual subject summaries)*.* Similarly, the performance of chimpanzees was not significantly different from chance at the group-level in the first 150 trials of Povinelli’s [12] weight sorting task (see data depicted in Fig 3) (model 3a; intercept estimate: 0.262, s.e. ± 0.257, z = 1.02, p = 0.309). The overall estimated probability of being correct for the group was 56.5%. The performance of one individual was statistically significantly above chance level (see Table C in Supporting information).

**Fig 4:**
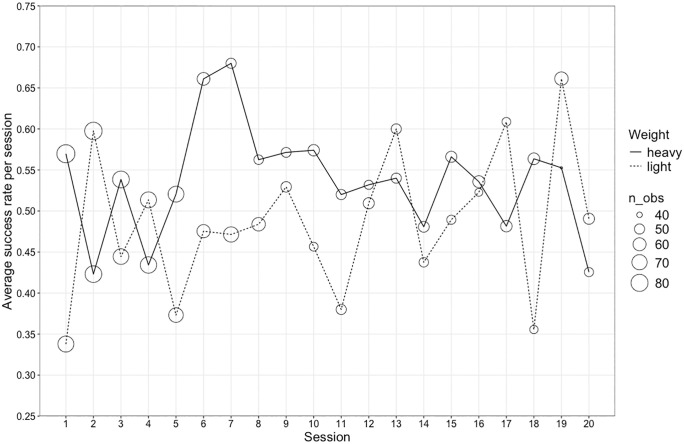
Goffin cockatoos’ average success rate by weight. The average success rate of all Goffin subjects each session of the sorting task, shown separately for trials with the heavy weight (unbroken line) and trials with the light weight (dotted line). The size of the circle represents how many observations contributed to the average value shown.

**Fig 5:**
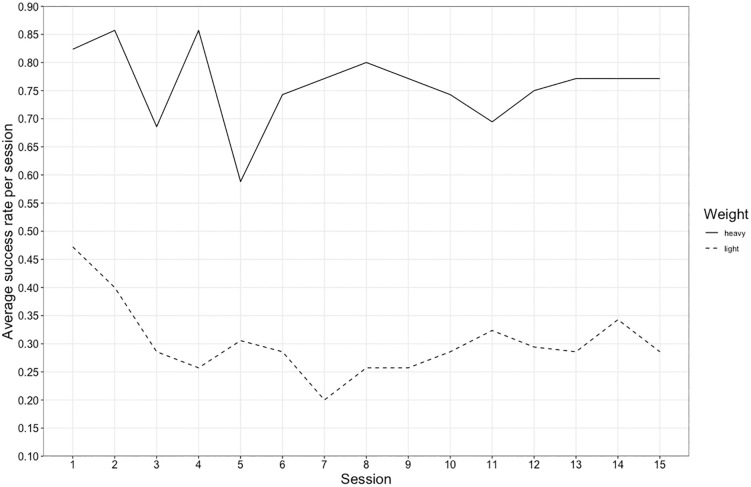
Chimpanzees’ average success rate by weight. The average success rate of all chimpanzee subjects each session of the sorting task, shown separately for trials with the heavy weight (unbroken line) and trials with the light weight (dotted line). Each individual (n = 7) participated in all trials so there is minimal variation in the number of observations (range 34–36) contributing to the average values shown.

There was no statistically significant effect of weight type on success levels in Goffin subjects (see group-level data depicted in Fig 4, and see Fig B in Supporting information for a break down by individual) (Model 1b; full-null model comparison: χ^2^ = 5.401, d.f. = 4, p = 0.249). Similarly, a full-null comparison also revealed no significant effect of weight type on success for chimpanzees in their first 15 sessions (see group-level data depicted in Fig 5, and see Fig F in Supporting information for a break down by individual) (model 3b; full-null model comparison: χ^2^ = 4.993, d.f. = 4, p = 0.288) (see Discussion).

**Fig 6:**
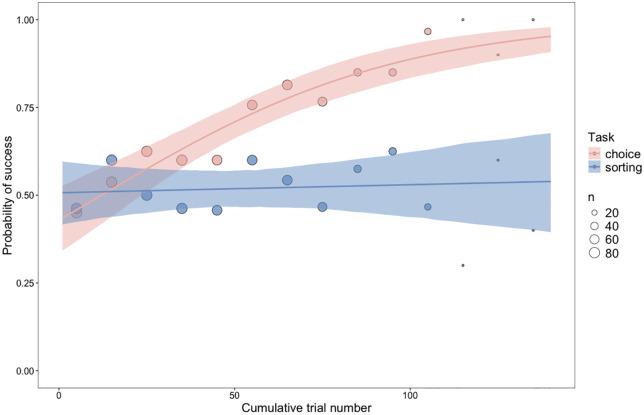
The influence of task on the probability of success. The influence of weight discrimination task (choice and sorting) on the probability of success in Goffin subjects (n = 8). The value of the points shows the averages of the raw data (for blocks of 10 trials) and the size of the point indicates the number of observations.

Lastly, the comparison of the performance levels of Goffin subjects in the weight choice and weight sorting discrimination tasks shows a significant effect of task (in interaction with trial number) (see Fig 6) (model 2; full-null model comparison: χ^2^ = 27.439, d.f. = 2, p < 0.001; z.cumul.trail:task estimate: −0.739, s.e. ± 0.157, z = −4.712, p < 0.001). Estimates from all models with the associated minimum, maximum and confidence interval values can be found in Tables S6–S17.

## Discussion

None of our subjects performed overall significantly above chance levels in our conditional discrimination task, where an object had to be sorted into the tray of the correct colour depending on whether it was heavy or light, over 200 trials. One signal of task difficulty can be biases in subject’s choices (e.g., [46,47]): in this task, some individuals exhibited a tendency to choose the tray on a particular side (see Figs D and E in Supporting information), or of a particular colour (see Fig C in Supporting information). This tendency could be relatively consistent or change directions across sessions. Our subjects have displayed biases previously on discrimination tasks where they failed to solve the tasks above chance levels (e.g., side biases: [28,38]; tool biases: [39]). We did not correct for their biases during testing in this case, as we were interested in how easy, or difficult, it was for our subjects to learn the task based upon weight cues, and individuals can overcome biases themselves when they start to attend to the right information for solving the task (see, e.g., the rats of [6]).

The performance of subjects provides no support for our hypothesis that Goffin’s cockatoos would be able to learn a conditional discrimination relatively quickly when it involves an ecologically relevant stimulus, namely weight. The two weights used in the sorting task were almost the same, at 3g and 23g, as the weights that our subjects have already shown that they can discriminate between [8]. Success in the present sorting task depended also on the ability of our subjects to discriminate between red and blue. We are confident that these two colours are discriminable for our subjects, primarily because birds are known to have excellent tetrachromatic colour vision [48], and further because some subjects even preferentially chose one colour tray over the other in some sessions. Therefore, we conclude that the poor performance of our subjects in the task presented here is much more likely to be due to cognitive factors, than limits on perception.

The low performance of our subjects on the weight sorting task is in contrast to the quick learning (an average of 60.6 trials to criterion) our subjects demonstrated on the previous weight discrimination task [8]; a within-individual difference in success rates that was statistically significant. The results from the two studies taken together provide further evidence in line with the understanding that conditional discriminations are more cognitively demanding than simple discriminations. However, there are other differences in task contingencies which may have also contributed to the difficulty of this weight sorting task, relative to the weight choice task [8], beyond the difference in discrimination formats. For example, in the weight choice task, the birds experienced the weight of both object types within a few seconds of each other. The temporal closeness of perception of the two weight types was an intended feature of the method designed by the authors. In contrast, it is possible that the lack of comparison of weight types within a trial in the weight sorting task made it more difficult for the birds to correctly identify which object they were carrying, as this would require memory of the experience of lifting (i.e., ‘effort-to-lift’ [12]) the other weight type in previous trials, and created the possibility of a time-error in discrimination (e.g., see [11]). In the weight choice task [8], there was in fact a positive effect of subjects switching between objects before making a choice. This behaviour was likely for the purpose of comparing the weight of the objects; we know that immediate comparisons between stimuli can accelerate the learning of a discrimination (e.g., [49]). Although we consider weight an ecologically relevant object property, another potential difficulty of the present task might lie in the requirement to build associations between cues belonging to two different modalities, weight (proprioceptive) information and colour (visual) information, which do not often need to be paired in an ecological context, or otherwise do not have systematic correspondences in nature [50].

We hypothesised that there might be a difference in success rate for our subjects in trials where the heavy or light weight was presented– i.e., that it would be easier for them to learn the sorting rule for one weight type over the other (see Introduction). Indeed, in their first weight discrimination task, Goffin subjects found it easier to learn the discrimination if they were rewarded for the light object [8, Tables S6 and S8] – a result also seen in rats [6]. When looking at the grouped data for chimpanzees in their weight sorting task [12], there is a higher success rate on trials with the heavy weight versus trials with the light weight (see Fig 5). However, in our analysis assessing the influence of weight type on success in our sorting study and Povinelli’s [12, Experiment 9] sorting study, there was no statistically significant effect of weight type for either species. This is likely due to the inter-individual variation in subjects’ responses (see Figs B and F in the Supporting information), which can also be linked, in the chimpanzees’ case, to differences in the direction of subject’s side biases (see Figs G and H and Table E in the Supporting information). In Goffin subjects who showed a divergence in success levels between the two weight types, such patterns could relate to a colour bias (see Fig C in the Supporting information). We have explored this possibility with the data presented in Table D (see Supporting information). In contrast to the sorting study with chimpanzees, a clear side bias in our subjects results in approximately equal success rates with the two weight types, given that the coloured trays moved between trials.

The results of our present study suggest that the previously indicated difference in the weight discrimination learning abilities demonstrated by our subjects [8] and the chimpanzees of Povinelli’s [12] study, is strongly affected by methodological task differences. On the basis of only the results we present here, it is possible that there may not be a marked difference between the two species with regards to this ability. However, there are slight differences in methodology between our present study and that of Povinelli’s that should be noted. For example, by randomizing the left-right positioning of the coloured trays over trials, we likely presented a more difficult task to the birds as they could only use or depend on one cue, colour, rather than two, location and colour, to learn where to place the different weight types. The lesser contrast of the two different weights in our study (a ratio of 1:5.75) compared to contrast of those used for the chimpanzees (1:9.6) is another methodological difference that might have made our task more difficult to learn, given that greater stimulus contrast – or lower stimulus similarity – is understood to accelerate learning (see [51]). Furthermore, because the sorting task was something Povinelli and his team wanted the chimpanzees to master as a basis for further experiments, they employed early orientation sessions, not included in the reported total number of trials taken to reach criterion, to help subjects initially learn the task’s contingencies. On the other hand, the fact that our subjects had previous experience with a weight-based discrimination might have increased their attention to weight information [52], or enhanced their ability to learn a rule involving an object’s weight, promoting learning in the second weight discrimination task. This relates to a more general point, with regards to a limitation of our work on our ability to understand the natural cognitive abilities of Goffin’s cockatoos: our subjects receive many different types of object-based enrichment in a non-experimental context, and several individuals already have years of experience with problem-solving experimental tasks, both which could have primed them for elevated levels of success on any physical learning task (although see [53] for a comparison of innovation rates between lab and wild-caught Goffin’s cockatoos). Lastly, whilst we note that we were able to test more than twice as many cockatoos as chimpanzees in [12], our sample size is likely small considering we would wish to fully represent the extent of between-individual variation in abilities in the species. These limitations are characteristic of cognition research with exotic animals in a lab context.

Overall, we can conclude from the performances of both species, Goffin’s cockatoos and chimpanzees, in the respective studies (and see also [5,9–11]) that building associations with the characteristic of weight – a feature only available whilst directly handling an object – is not necessarily an easy task for an animal, although this does depend on the method employed. Discrimination tasks are often used to ascertain whether a species can perceive a particular object feature or information in a particular modality. However, a failure to learn a discrimination does not mean that the species cannot perceive the characteristic in question– the contrasting performances of our subjects between the two tasks is a case in point. It can be instead that subjects have not been able to learn which of the stimulus features are relevant to the task [54], for example colour not location as in our study, or that the learning requirements of the task go beyond any naturalistic situation that the species has evolved to cope with. Following on from our work, we suggest that conditional discrimination tasks, with the greater cognitive demands they pose, might not the best method for comparative questions related to perception or sensitivity (i.e., attention) to particular object properties in different species.

Lastly, if only considering the performances of species on simple weight discrimination tasks, Goffin’s cockatoos mastered a weight-based discrimination much more quickly than other primates tested with the same type of discrimination format (with alternative methods) [9–11]. To answer the question of whether there is a real difference in this cognitive ability between avian and primate species, we suggest future research should replicate a simple discrimination task between the groups so that a firmer conclusion about the evolution of the cognitive ability can be drawn. We further suggest that in any future studies not only task contingencies should be considered, but also stimulus salience given that the influence of this feature of discrimination learning with weight has thus far been mostly overlooked. In fact, Macphail’s ‘Null Hypothesis’ [55] posits that any apparent difference between nonhuman animals in cognitive capacities can actually be explained by contextual variables, such as how stimuli are presented, or differences in how species perceive them. Whilst this view is somewhat extreme according to contemporary research (see [56]), the comparative question of the perception of weight is undoubtedly made more difficult, and more interesting, when comparing species possessing very different body sizes and morphologies, such as beaks and hands. A promising future direction would be to investigate if and how categorically different morphologies might affect how weight is perceived.

## Supporting information

S1 File Further details on methods, analysis and descriptive results. (PDF)

S2 File S8–S17 Tables. Model estimates. (XLSX)

S1 R Script R Script for analysis. (R)

S1 Dataset Goffin sorting task dataset. (CSV)

S2 Dataset Goffin task comparison dataset. (CSV)

S3 Dataset Chimpanzee sorting task dataset. (CSV)

S1 Video Video of a correct and incorrect trial in the Goffin sorting task. (MP4)
