# Supplementary material for: Testing weight-based conditional discrimination in Goffin’s cockatoos, Cacatua goffiniana
Source: PLoS One. 2025 Dec 19;20(12):e0338604. doi: 10.1371/journal.pone.0338604 (PMC12716734; doi:10.1371/journal.pone.0338604)
Supplement: S1 File — (PDF) [file pone.0338604.s001.pdf]

Testing weight-based conditional discrimination in Goffin's cockatoos, *Cacatua goffiniana*

Poppy J. Lambert, Antonia Rippel-Rachle & Alice M. I. Auersperg

SUPPORTING INFORMATION

Contents

Supporting information: Methods…………………….....p. 2
Table A……………………………………………..…p. 3
Table B……………………………………………..…p. 4
Table C……………………………………………..…p. 5
Figure A…………………………………………….p. 6
Figure B…………………………………………….p. 7
Table D……………………………………………..…p. 8
Figure C…………………………………………….p. 9
Figure D…………………………………………….p. 10
Figure E…………………………………………….p. 11
Figure F…………………………………………….p. 12
Table E……………………………………………….p. 13
Figure G…………………………………………….p. 14
Figure H…………………………………………….p. 15
Supporting information: Statistical analysis…………..p. 16
References…………………………………………..p. 17

Testing weight-based conditional discrimination in Goffin's cockatoos, *Cacatua goffiniana*

Poppy J. Lambert, Antonia Rippel-Rachle & Alice M. I. Auersperg

SUPPORTING INFORMATION

Supporting information: Methods

Procedure: weight and tray position combinations

The four possible combinations of weight type and tray placement (heavy object -blue tray left, heavy object -blue tray right, light object -blue tray left, light object -blue tray right) for each trial was pseudorandomised for each bird individually, on the condition that a single combination didn't occur more than three times in row within a session. The combination used each trial is recorded in the included data set. Each individual received approximately the same numbers of each trial type during their testing.

Testing weight-based conditional discrimination in Goffin's cockatoos, *Cacatua goffiniana*

Poppy J. Lambert, Antonia Rippel-Rachle & Alice M. I. Auersperg

SUPPORTING INFORMATION

Table A

	**Group 1** *(light -> red, heavy -> blue)*		**Group 2** *(light -> blue, heavy -> red)*	
	**Male**	**Female**	**Male**	**Female**
**Adult**	Pipin, Kiwi, Zozo, Konrad	Olympia, Fini, Mayday	Muppet, Figaro, Dolittle, Muki	Heidi, Moneypenny
**Subadult**		Jane	Titus	Irene

Table A. Subjects categorised according to age (adult/subadult), sex and group. Group 1 subjects were rewarded for placing the light object into the red tray and the heavy object into the blue tray. Group 2 subjects were rewarded for placing the heavy object into the red tray and the light object into the blue tray.

Poppy J. Lambert, Antonia Rippel-Rachle & Alice M. I. Auersperg

SUPPORTING INFORMATION

Table B

Subject (Goffin)	Age group	Overall success rate (% trials correct) of subjects in the weight sorting task
Jane	Subadult	44.5
Muki	Adult	46.0
Fini	Adult	48.0
Zozo	Adult	48.0
Konrad	Adult	48.6
Figaro	Adult	50.0
Heidi	Adult	50.0
Irene	Subadult	50.0
Moneypenny	Adult	50.0
Titus	Subadult	50.0
Mayday	Adult	51.5
Olympia	Adult	52.0
Dolittle	Adult	52.5
Pipin	Adult	54.5
Muppet	Adult	55.5
Kiwi	Adult	57.0

Poppy J. Lambert, Antonia Rippel-Rachle & Alice M. I. Auersperg

SUPPORTING INFORMATION

Table C

**Subject (chimpanzee)**	**Overall success rate (% trials correct) of subjects in the weight sorting task**
Mindy	46.0
Kara	48.0
Jadine	48.7
Apollo	54.0
Candy	54.0
Megan	54.7
Brandy*	66.7

*The performance of this individual (100 success out of 150 trials) is statistically significantly above chance level (exact binomal test, alternative hypothesis = 'greater'; p-value < 0.001).

Testing weight-based conditional discrimination in Goffin's cockatoos, *Cacatua goffiniana*

Poppy J. Lambert, Antonia Rippel-Rachle & Alice M. I. Auersperg

SUPPORTING INFORMATION

Figure A

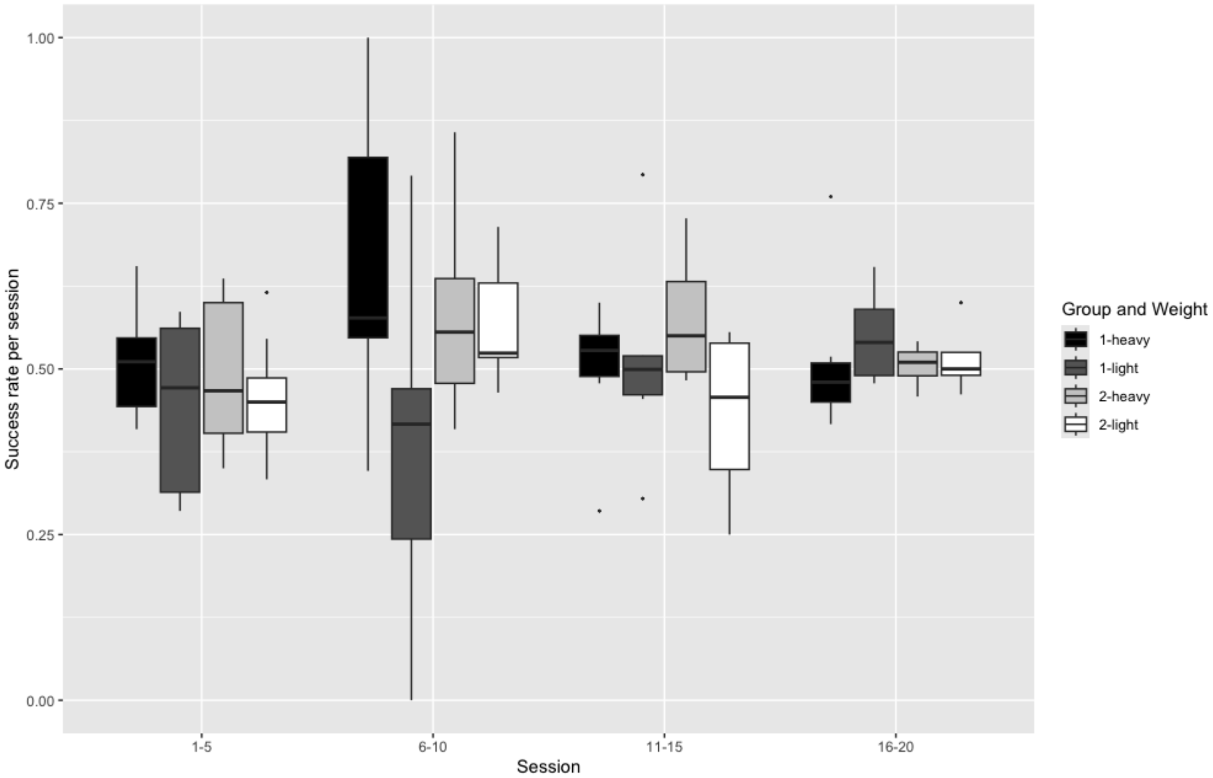

Figure A. Success rate for Goffin subjects in group 1 and group 2, summarised for light and heavy trials separately over blocks of five sessions. Each observation is the average success rate of an individual on heavy/light trials over all five sessions in that block.

Testing weight-based conditional discrimination in Goffin's cockatoos, *Cacatua goffiniana*

Poppy J. Lambert, Antonia Rippel-Rachle & Alice M. I. Auersperg

SUPPORTING INFORMATION

Figure B

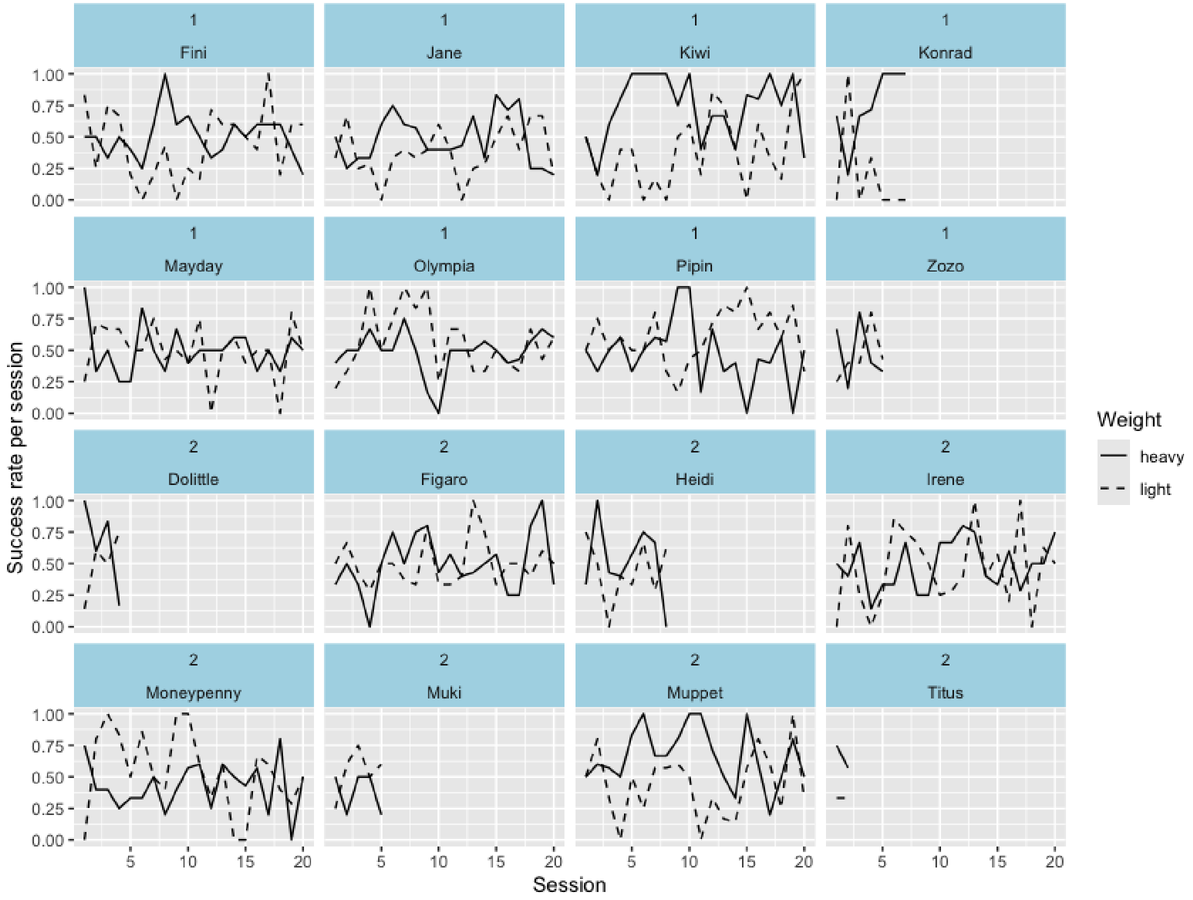

Figure B. Individual Goffin success rate on heavy and light trials each session. Subjects are shown in the two groups (group 1 = top two rows, group 2 = bottom two rows).

Testing weight-based conditional discrimination in Goffin's cockatoos, *Cacatua goffiniana*

Poppy J. Lambert, Antonia Rippel-Rachle & Alice M. I. Auersperg

SUPPORTING INFORMATION

Table D

**Subject (Goffin)**	**Group** (group and sorting rule: 1 = heavy ->blue; 2 = light ->blue)	**Overall success rate (% trials correct) with the light weight**	**Overall success rate (% trials correct) with the heavy weight**	**Colour bias: overall percentage blue tray chosen**
Dolittle	2	45.0	60.0	42.5
Figaro	2	49.0	51.0	49.0
Fini	1	47.0	49.0	51.5
Heidi	2	47.6	52.6	47.5
Irene	2	51.5	48.5	51.5
Jane	1	39.0	50.0	54.5
Kiwi	1	41.0	73.0	65.5
Konrad	1	18.8	73.7	77.1
Mayday	1	51.0	52.0	50.5
Moneypenny	2	55.4	44.4	55.0
Muki	2	54.5	39.3	56.0
Muppet	2	45.1	66.3	39.5
Olympia	1	56.6	47.5	45.5
Pipin	1	62.1	46.4	42.5
Titus	2	33.3	63.6	40.0
Zozo	1	46.2	50.0	52.0

Testing weight-based conditional discrimination in Goffin's cockatoos, *Cacatua goffiniana*

Poppy J. Lambert, Antonia Rippel-Rachle & Alice M. I. Auersperg

SUPPORTING INFORMATION

Figure C

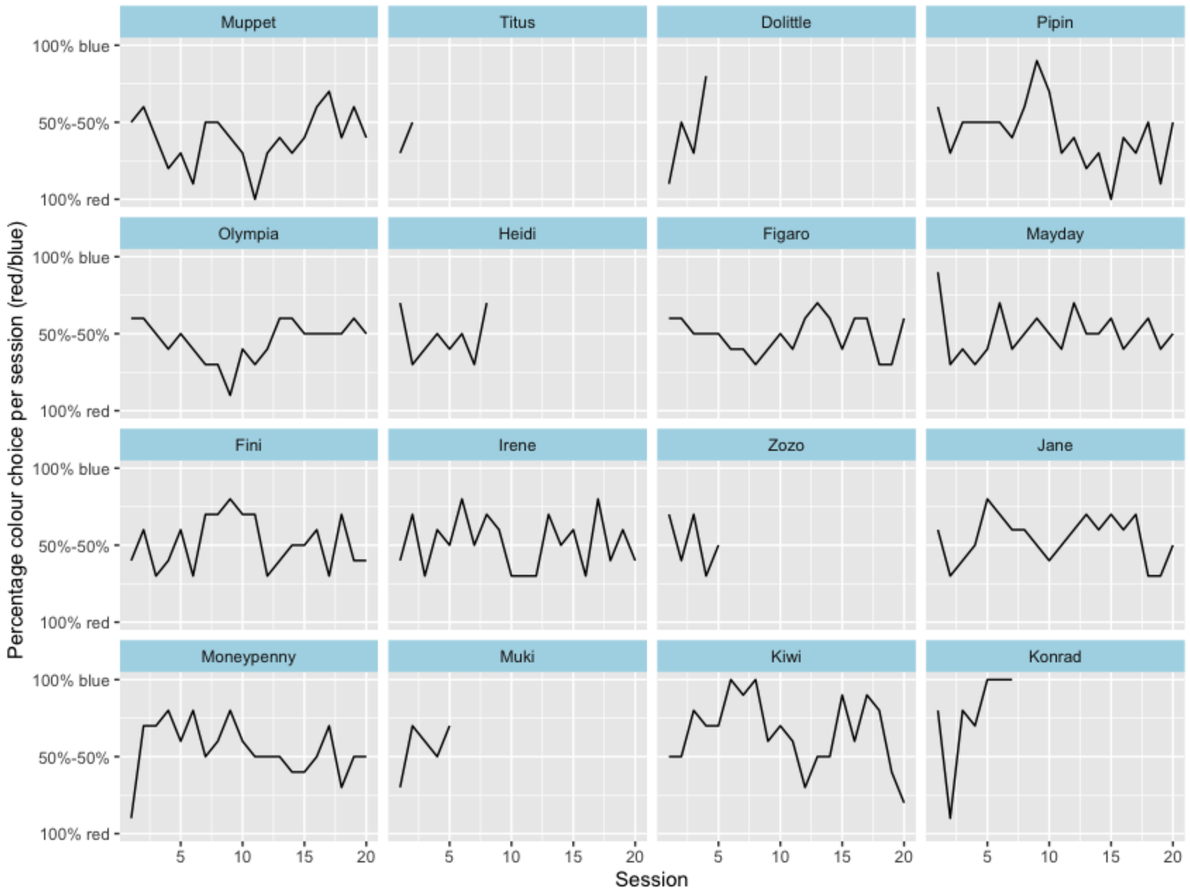

Figure C. The percentage of trials each session Goffin subjects chose to place the weighted object in either the red or blue tray. Individuals are organised from lowest overall percentage of choices blue (i.e. most red)(top left) through to highest overall percentage of choices blue (bottom right).

Testing weight-based conditional discrimination in Goffin's cockatoos, *Cacatua goffiniana*

Poppy J. Lambert, Antonia Rippel-Rachle & Alice M. I. Auersperg

SUPPORTING INFORMATION

Figure D

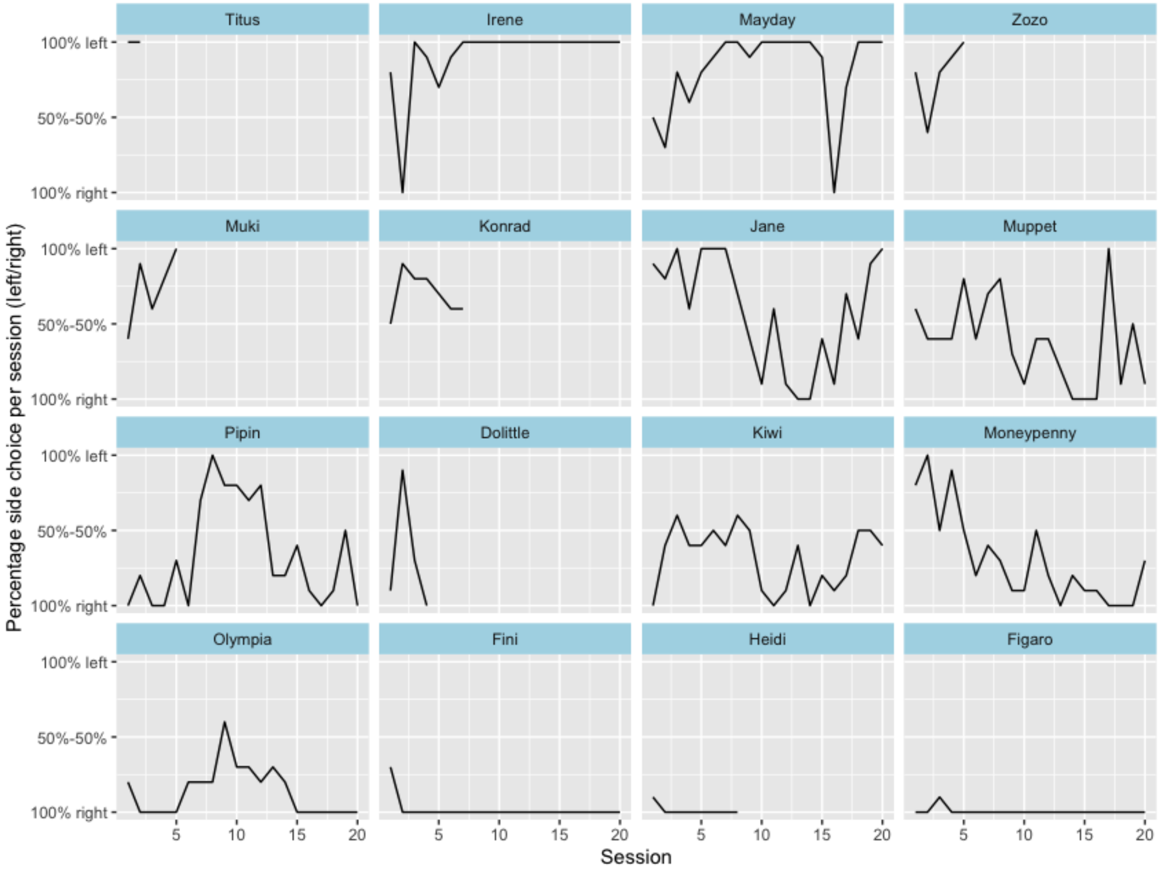

Figure D. The percentage of trials each session Goffin subjects chose the tray on the left or right. Individuals are organised from highest overall percentage of choices left (top left) through to lowest overall percentage of choices left (i.e. most overall right) (bottom right).

Testing weight-based conditional discrimination in Goffin's cockatoos, *Cacatua goffiniana*

Poppy J. Lambert, Antonia Rippel-Rachle & Alice M. I. Auersperg

SUPPORTING INFORMATION

Figure E

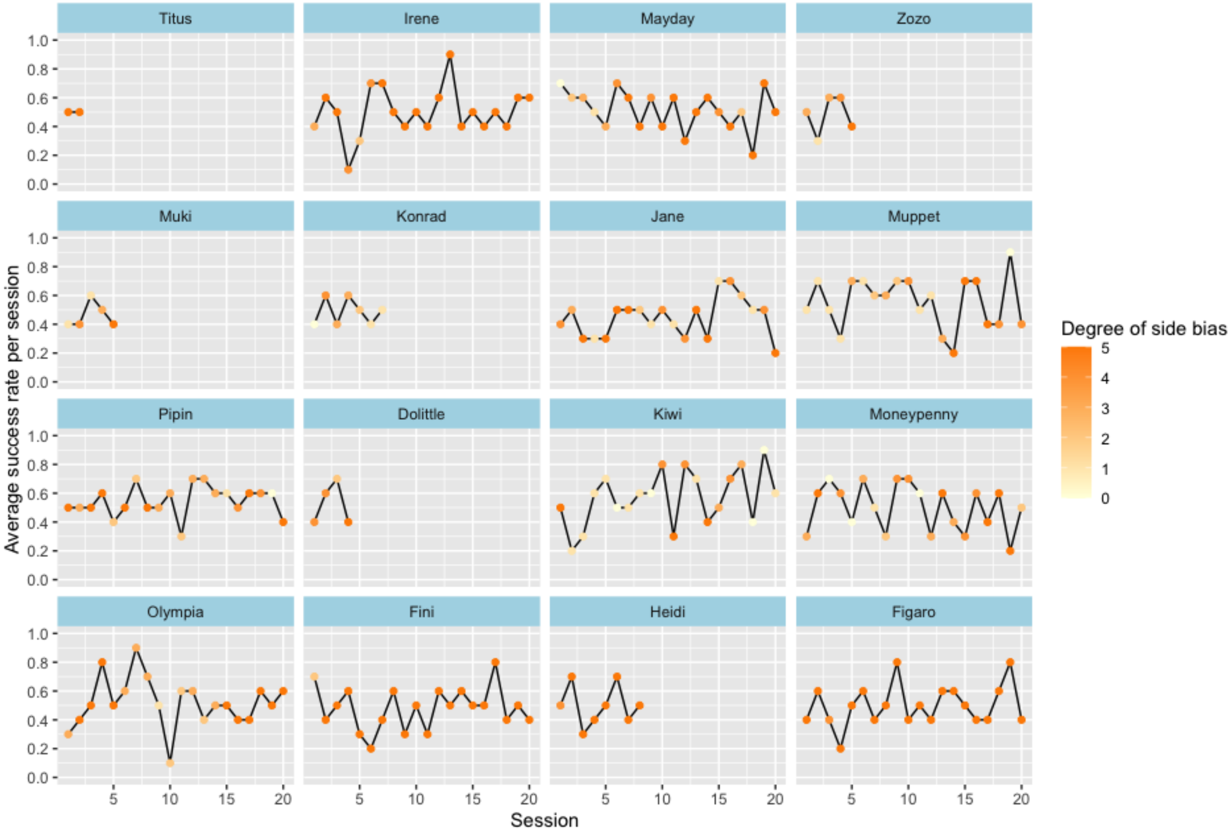

Figure E. Individual Goffin success rate each session, where the degree of side bias (0 = a 50:50 split in choice of side, 5 = 100% one side chosen) is indicated by the colour of the point. Individuals are organised in order of most left biased through to most right biased, i.e. the more extremely biased individuals are found in the top left and bottom right.

Testing weight-based conditional discrimination in Goffin's cockatoos, *Cacatua goffiniana*

Poppy J. Lambert, Antonia Rippel-Rachle & Alice M. I. Auersperg

SUPPORTING INFORMATION

Figure F

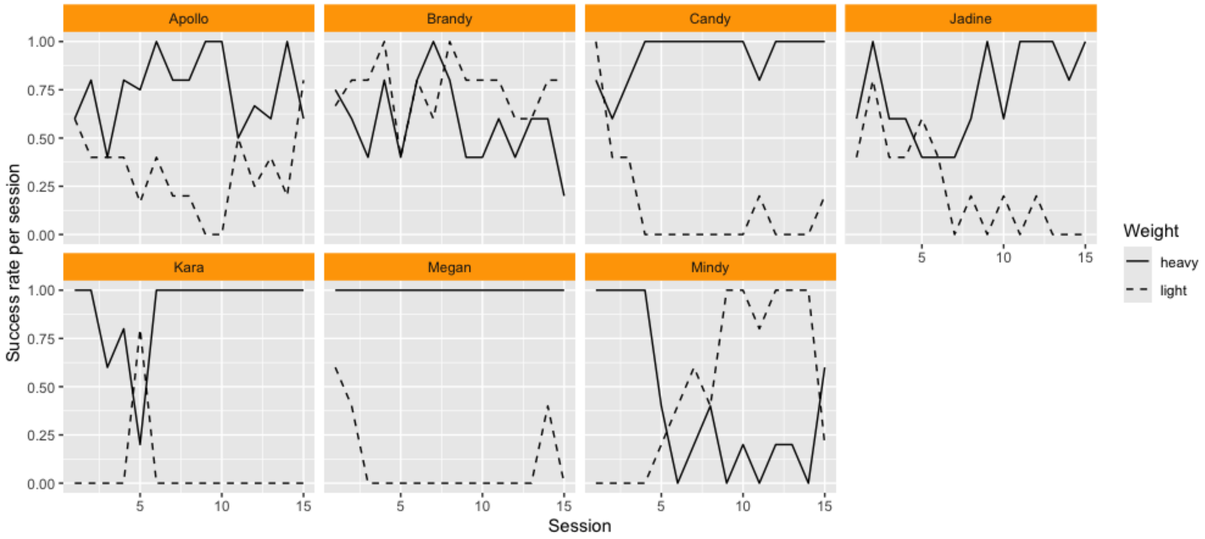

Figure F. Individual chimpanzee success rate on heavy and light trials each session.

Testing weight-based conditional discrimination in Goffin's cockatoos, *Cacatua goffiniana*

Poppy J. Lambert, Antonia Rippel-Rachle & Alice M. I. Auersperg

SUPPORTING INFORMATION

Table E

**Subject (chimpanzee)** (sorting rule: heavy -> blue/right)	**Overall success rate (% trials correct) on trials with the <u>light</u> weight**	**Overall success rate (% trials correct) on trials with the <u>heavy</u> weight**	**Side bias: overall percentage right/blue tray chosen**
Apollo	32.4	75.0	71.3
Brandy	75.0	58.1	41.3
Candy	14.7	93.3	89.3
Jadine	24.0	73.3	74.7
Kara	5.3	90.7	92.7
Megan	9.3	100	95.3
Mindy	50.7	41.3	45.3

Testing weight-based conditional discrimination in Goffin's cockatoos, *Cacatua goffiniana*

Poppy J. Lambert, Antonia Rippel-Rachle & Alice M. I. Auersperg

SUPPORTING INFORMATION

Figure G

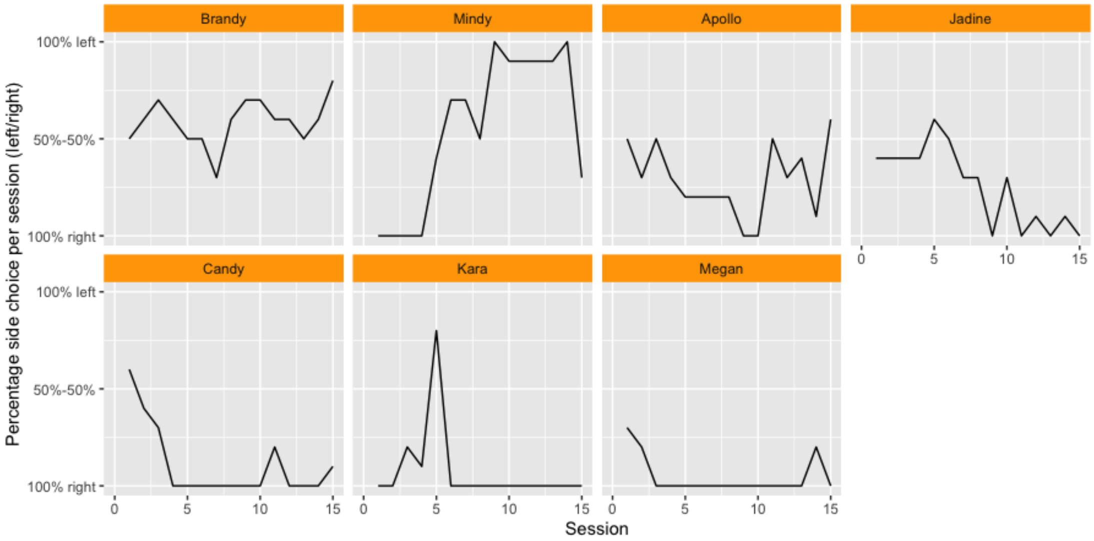

Figure G. The percentage of trials each session chimpanzee subjects chose the tray on the left or right. Individuals are organised from highest overall percentage of choices left (top left) through to lowest overall percentage of choices left (i.e. most overall right) (bottom most right).

Testing weight-based conditional discrimination in Goffin's cockatoos, *Cacatua goffiniana*

Poppy J. Lambert, Antonia Rippel-Rachle & Alice M. I. Auersperg

SUPPORTING INFORMATION

Figure H

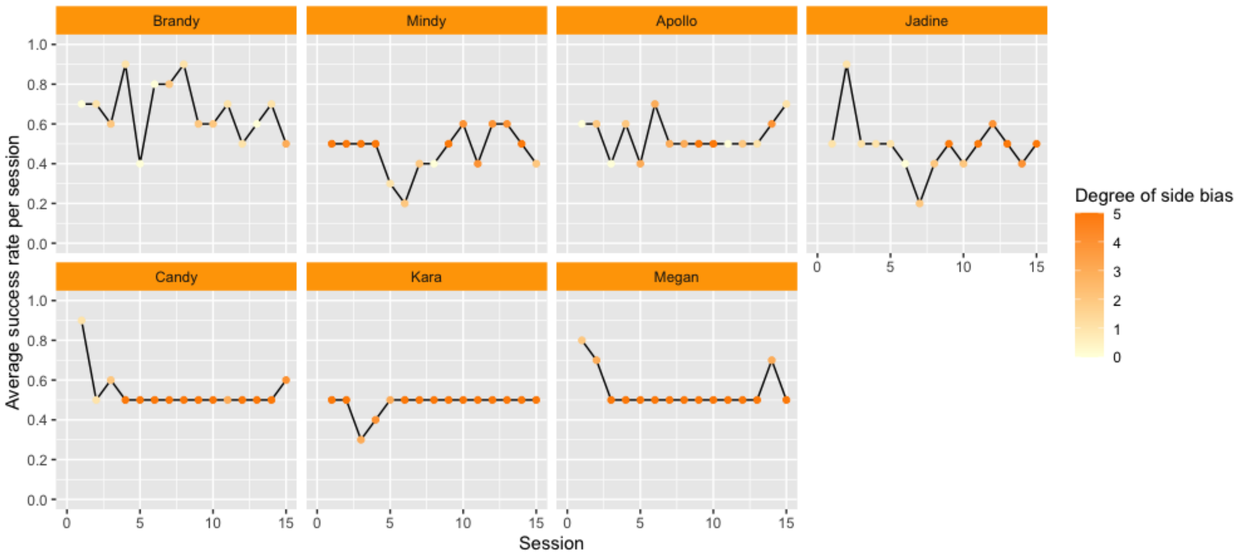

Figure H. Individual chimpanzee success rate each session, where the degree of side bias (0 = a 50:50 split in choice of side, 5 = 100% one side chosen) is indicated by the colour of the point. Individuals are organised in order of most left biased through to most right biased, i.e., the more extremely biased individuals are found in the top left and bottom right.

Testing weight-based conditional discrimination in Goffin's cockatoos, *Cacatua goffiniana*

Poppy J. Lambert, Antonia Rippel-Rachle & Alice M. I. Auersperg

SUPPORTING INFORMATION

Supporting information: Statistical analysis

Testing assumptions and model stability

After fitting each model we confirmed that none of the model assumptions were violated: we calculated 'Variance Inflation Factor' (VIF) (Field, 2005) to ensure that collinearity was not an issue (max VIF = 1.007) and visually inspected whether 'Best Linear Unbiased Predictors' (BLUPs) were approximately normally distributed (Baayen, 2008; Harrison et al., 2018). To assess the stability of each of the three models, we compared estimates from the models based on all the data with the estimates obtained from models where each of the levels of random effects were excluded one at a time (Nieuwenhuis *et al.*, 2012). This showed us that the stability of models 1a/b and 3a/b were moderate with regards to the weight/weight.code estimate (and also informed our decision to include no correlations between random intercepts and slopes in model 2).

Model implementation

All statistical analyses were performed in R-Studio (RStudio Team; version: 2024.9.1.394) using the open-source software R (R Core Team; version: 4.4.2). Generalized Linear Mixed Models (GLMM) were fitted using the function 'glmer' (Bates *et al.*, 2015; package 'lme4'; version: 1.1-35.5). We used the optimizer 'bobyqa' and set iterations to 1,000,000 for all models. To assess 'Variance Inflation Factors' we used the function 'vif' of the package 'car' (Fox & Weisberg, 2019; version: 3.1-3). We inspected BLUPs with a package kindly provided by Roger Mundry. We assessed model stability and calculated confidence intervals using functions also provided by Roger Mundry. Confidence intervals were derived by applying the function 'bootMer' of the package 'lme4', using 1,000 parametric bootstraps for both fixed effects and random effects. The function 'anova' (argument 'test' set to 'Chisq') was used for likelihood ratio tests (full-null comparison).

Testing weight-based conditional discrimination in Goffin's cockatoos, *Cacatua goffiniana*

Poppy J. Lambert, Antonia Rippel-Rachle & Alice M. I. Auersperg

SUPPORTING INFORMATION

References

Baayen, R. (2008). *A practical introduction to statistics using R. Analyzing Linguistic Data*. Cambridge University Press.

Bates, D., Mächler, M., Bolker, B., & Walker, S. (2015). *Fitting linear mixed-effects models using lme4*. *67*(1), 1–48.

Field, A. (2005). *Discovering statistics using SPSS. Thousand Oaks, CA, US*.

Fox, J., & Weisberg, S. (2019). *An R Companion to Applied Regression (Third Edition). R package version 3.0*.

Harrison, X. A., Donaldson, L., Correa-Cano, M. E., Evans, J., Fisher, D. N., Goodwin, C. E., Robinson, B. S., Hodgson, D. J., & Inger, R. (2018). A brief introduction to mixed effects modelling and multi-model inference in ecology. *PeerJ*, *6*, e4794.

Nieuwenhuis, R., Grotenhuis, M., & Pelzer, B. (2012). *influence.me: Tools for detecting influential data in mixed effect models*. *4*, 38–47.

R Core Team (2024). R: A Language and Environment for Statistical Computing. R Foundation for Statistical Computing, Vienna, Austria.

Posit team (2024). RStudio: Integrated Development Environment for R. Posit Software, PBC, Boston, MA.
